# Investigation of Indoor Air Quality in Houses of Macedonia

**DOI:** 10.3390/ijerph14010037

**Published:** 2017-01-01

**Authors:** Silvia Vilčeková, Ilija Zoran Apostoloski, Ľudmila Mečiarová, Eva Krídlová Burdová, Jozef Kiseľák

**Affiliations:** 1 Institute of Environmental Engineering, Faculty of Civil Engineering, Technical University of Košice, Vysokoškolská 4, 04200 Košice, Slovakia; eko-inzenering@t-home.mk (I.Z.A.); ludmila.meciarova@tuke.sk (Ľ.M.); eva.kridlova.burdova@tuke.sk (E.K.B.); 2 Institute of Mathematics, Faculty of Science, Pavol Jozef Šafárik University in Košice, Jesenná 5, 04001 Košice, Slovakia; jozef.kiselak@upjs.sk

**Keywords:** family house, temperature, humidity, TVOC, PM, sound pressure level, statistical analysis

## Abstract

People who live in buildings are exposed to harmful effects of indoor air pollution for many years. Therefore, our research is aimed to investigate the indoor air quality in family houses. The measurements of indoor air temperature, relative humidity, total volatile organic compounds (TVOC), particulate matters (PM) and sound pressure level were carried out in 25 houses in several cities of the Republic of Macedonia. Mean values of indoor air temperature and relative humidity ranged from 18.9 °C to 25.6 °C and from 34.1% to 68.0%, respectively. With regard to TVOC, it can be stated that excessive occurrence was recorded. Mean values ranged from 50 μg/m^3^ to 2610 μg/m^3^. Recommended value (200 μg/m^3^) for human exposure to TVOC was exceeded in 32% of houses. Mean concentrations of PM_2.5_ (particular matter with diameter less than 2.5 µm) and PM_10_ (diameter less than 10 µm) are determined to be from 16.80 µg/m^3^ to 30.70 µg/m^3^ and from 38.30 µg/m^3^ to 74.60 µg/m^3^ individually. Mean values of sound pressure level ranged from 29.8 dB(A) to 50.6 dB(A). Dependence between characteristics of buildings (Year of construction, Year of renovation, Smoke and Heating system) and data from measurements (Temperature, Relative humidity, TVOC, PM_2.5_ and PM_10_) were analyzed using R software. Van der Waerden test shows dependence of Smoke on TVOC and PM_2.5_. Permutational multivariate analysis of variance shows the effect of interaction of Renovation and Smoke.

## 1. Introduction

Indoor environmental quality (IEQ) is an essential condition to establish a healthy housing environment [1] and is crucially linked to occupant′s health and well-being [2]. Human beings have endeavored to create indoor environments in which they can feel comfortable. Human health is foremost when it comes to assessing the overall comfort of the environment. If the built environment is leading to sickness or negative impact on the occupant′s health for any reason then it could lead to some design or technical flaw in the building system [3]. Building structures are linked with a range of health hazard, such as those attributable to extreme temperatures, indoor air pollution, noise, airborne infectious diseases or mold contamination [4]. Today, there is no secret that long-term as well as short-term exposure to PM_2.5_ has been associated with increased respiratory and cardiovascular morbidity [5]. Study [6] showed that major contributor to total indoor volatile organic compounds in residences were households products, followed by combustion processes and environmental tobacco smoke, deodorizers and off-gassing of building materials. These chemicals can cause irritation of eyes or nose, dizziness, nausea, headaches and allergic reactions and some of them are carcinogenic [7,8]. Deng et al. [9] revealed that exposure to new furniture and home redecoration during pregnancy significantly increased childhood asthma. The knowledge of IEQ is useful for rehabilitation of buildings. It can indicate areas which require improvement in measures of the parameters which affect the indoor environment [10]. Nowadays many investigations are interested in the link between indoor environmental parameters, health effects and the well-being of the occupants as well as the implementation of sustainable practices in the building′s design and operation. Zalejska-Jonsson and Wilhelmsson [11] found that generally satisfaction with air quality has the highest impact on occupants′ overall satisfaction. Study [12] states that environmental factors such as thermal comfort, indoor air quality, aural and visual environments have notable combination of effects on the occupant′s acceptability and work performance. Further study [13] shows that a good understanding of IEQ issues in naturally ventilated buildings and the association with urban microclimate is fundamental for improving their IEQ. All over the world research teams investigates IEQ to find the main sources of pollution, to avoid indoor air pollution by designing optimal measures in existing building and by designing materials and furniture with zero emission; thus, ensuring high quality of indoor living conditions. Study [14] assesses IEQ in existing multi-family buildings in North–East Europe. Sixteen existing multi-family buildings (94 apartments) in Finland and 20 (96 apartments) in Lithuania were investigated prior to their renovation in order to assess the potential for improving IEQ along with energy efficiency. Data on temperature, relative humidity, CO_2_, CO, particulate matter, NO_2_, formaldehyde, volatile organic compounds, radon, and microbial content in settled dust were collected from each apartment. Results show substantial differences in indoor environmental conditions which were observed between the two countries. According to the research work [15], which was conducted in 32 typical residential apartments in Hong Kong, the investigated factors such as operative temperature, CO_2_ concentration, equivalent noise level and illumination level all had important effects on the overall IEQ acceptance. Thermal and aural environmental qualities were deemed the most important contributors, whereas indoor air quality was considered the least important. Another survey [16] was performed with a sample of 482 residents in high-rise residential buildings to investigate the impact of aspects of IEQ on occupant’s overall environmental satisfaction (OES). Results show that most of the items had significant impact on the occupant′s feelings regarding sub-factors. Adaptive behavior of the shading and lighting effect, the luminous comfort significantly and actively intensifies the mental stress; this has influence on the acoustic comfort the most. The study [17] summarizes the results of IAQ study over the following key findings: (a) negative relationship between the flat′s age and the selected indoor air pollutants; (b) combustion and cooking activities affect multiple types including CO, CO_2_, NO_2_, H_2_O and particles, and (c) no single activity emerges that controls indoor air quality. The study [18] concludes that a general decrease in indoor TVOC concentrations may be caused by the age difference of the buildings. Besides, it suggests that concentrations of TVOC tend to be lower in more recently built homes. According to many researches concentrated on health effects they cause serious problems. Review [19] focused on a summarized existing epidemiological evidence of the association between quantitative estimates of indoor air pollution with early childhood respiratory diseases; stated association between domestic exposure to VOCs and asthma in young children. Another study [20] states that adjusted odds ratio per 100 ppb increase in indoor TVOCs were slightly significant for upper respiratory syndrome, stuffy nose, dry throat and lower respiratory syndrome, non-specific syndrome, tiredness, anger and dizziness. Finally, the study [21] states that if a building is designed and constructed to be run, operated and occupied by people, then the requirements for their occupancy must be made as a prerequisite for their comfort. So, the significance of sustaining better IEQ in buildings should be a concern for both planners and managers of the built environment. It can be said as the study [22] notes that many of today′s sustainable building designs take the issue of IEQ.

The aim of the presented study is to analyze the indoor air quality of selected houses in Prilep, Macedonia. Indoor air factors such as indoor air temperature, relative humidity, total volatile organic compounds, particulate matters and sound pressure level were measured in 25 houses. The main contribution of the study is underlining the level of IAQ as well as finding dependence between building characteristics and measured indoor environmental parameters.

## 2. Materials and Methods

### 2.1. Selection of Objects

The research object is 25 homes situated in the Republic of Macedonia. Houses included in the IAQ study were selected from the area of south-western part of the Republic of Macedonia about 120 km from Skopje, in town Prilep. The details of the houses are shown in the Table 1.

Selecting of activities which were considered in statistical analysis related with occurrence of indoor air pollutants. There are scientific evidence that smoking has impact to occurrence of particulate matters and VOCs (e.g., benzene, ethylbenzene, and styrene) [23,24,25,26]. Renovation (thermal insulation of buildings) and year of construction affects indoor air temperature and relative humidity as well as concentrations of pollutants [27]. Concentrations of particulate matters can be also influenced by type of heating system [28,29]. The following building characteristics were chosen for the analysis: type of building—single-family houses and apartments in multi-family buildings; age of the building (construction finished in years from 1960 to 2005); renovation, smoking, and heating system.

### 2.2. Measurements of Indoor Air Quality Factors

Indoor air temperature, relative humidity, sound pressure level, particulate matters and total volatile organic compounds were measured in the selected family houses in the period from December 2012 until March 2013. In this period, the outside air temperature was in the range of −10 °C to 10 °C and the external humidity from 30% to 79%.

Indoor air temperature and relative humidity were measured by a temperature and humidity meter (Table 2, No. 1–2).

Total volatile organic compounds were measured with gas detector used advanced PID (photoionization detector) sensor. Gas detector can continuous detect aromatic hydrocarbons, unsaturated hydrocarbons, ketones, alcohols as well as aldehydes (Table 2, No. 3).

Particulate matters were measured by the nephelometer, which continuously indicates the concentration of thoracic, inhalable and respirable particles down to 0.1 µg/m^3^. In environmental mode, it indicates TSP, PM_10_, PM_2.5_ and PM_1_ concentrations. A pump continuously draws an air sample through the nephelometer which analyses the individual particles as they pass through a laser beam (Table 2, No. 4).

Noise level was determined by using sound level meter (Table 2, No. 5).

The data from all the instruments was downloaded to a computer for further analysis. During the measurements all of the instruments were placed approximately in the middle of the living room in the height of 1.1 m above the floor. The measurement lasted for 1 hour and 30 min during normal operation of the building. Each measurement was repeated three times. Living rooms were selected as reference rooms because building users spend substantial part of day in these spaces. The doors and the windows were closed throughout the measurement. More data concerning the instruments used is shown in Table 2.

### 2.3. Statistical Analyses

Characteristics of buildings (Year of construction, Year of renovation, Smoke and Heating system) and data from measurements (Temperature, Relative humidity, TVOC, PM_2.5_ and PM_10_) were used for statistical analysis using R software (R Foundation for Statistical Computing, Vienna, Austria, version 3.2.5) [30]. Since Normality property is violated we focused on nonparametric methods. Permutational multivariate analysis of variance (PERMANOVA, [31]) was used for multivariate analysis to determine the impact of continuous and categorical factors on measurements. To test null hypothesis (“The centroids of the groups, as defined in the space of the chosen resemblance measure, are equivalent for all groups.”), a pseudo-F-statistic, modelled on the classical F-statistic used in classical Analysis of variance (ANOVA), is constructed directly from the dissimilarity values in the matrix [32]. Important fact is that PERMANOVA is quite robust to correlations and heterogeneous variances. The PERMANOVA used the “adonis” procedure in the vegan package [33]. Ordinations were plotted with non-metric multidimensional scaling (NMDS) [34]; using the default settings of vegan′s “metaMDS” procedure and “ordiellipse”, which adds ellipses enclosing all points in the group (ellipsoid hulls) or ellipses of standard deviation, standard error or confidence areas. This allows us to visualize the level of similarity of individual cases of a dataset. It uses adequate dissimilarity measures, whereas standard distance on real line was chosen. Consequently, Van der Waerden normal scores test in the PMCMR package was used for univariate cases [35]. The advantage of the Van Der Waerden test is that it provides the high efficiency of the standard ANOVA analysis when the normality assumptions are in fact satisfied, but it also provides the robustness of the Kruskal-Wallis test when the normality assumptions are not satisfied.

## 3. Results and Discussion

**Figure 1:**
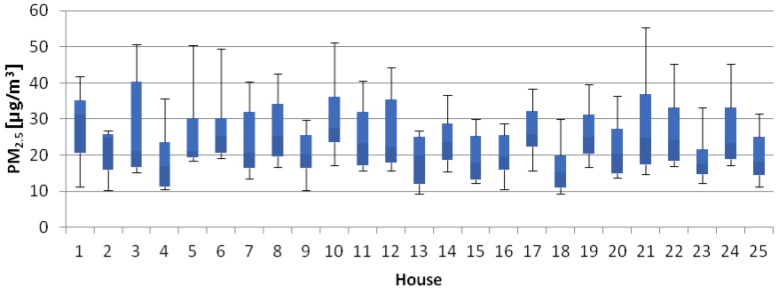
Box plot of PM_2.5_ concentrations.

**Figure 2:**
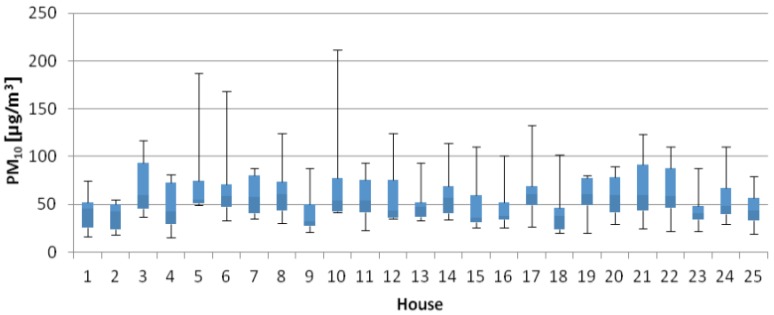
Box plot of PM_10_ concentrations.

**Figure 3:**
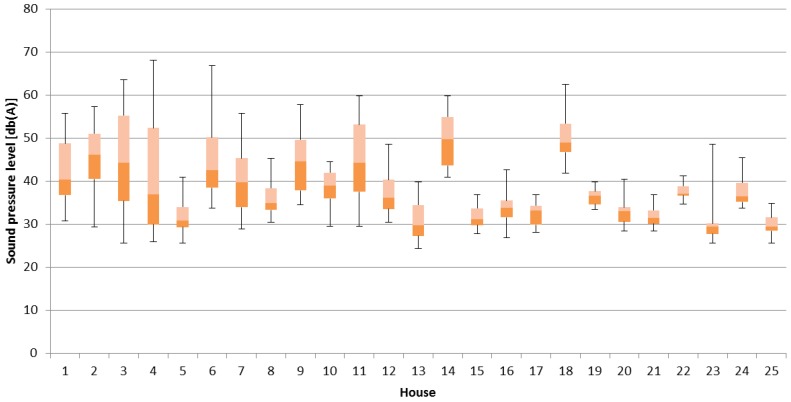
Box plot of sound pressure level.

The following table (Table 3) and figures (Figure 1, Figure 2 and Figure 3) present the results from the investigated indoor climate parameters. The bottom and the top of the boxes represent 25 and 75 percent and the band near the middle of the box is the median.

### 3.1. Temperature and Relative Humidity

The mean indoor air temperature ranges from 19.3 °C to 25.6 °C in single family houses and from 18.9 °C to 25.1 °C in apartments. Standard deviation (S.D.) ranges from 0.4 to 1.0. Similar values were found in study [18]. The mean indoor temperature set of 157 single-family houses and 148 apartments was found to be 21.4 °C and 22.5 °C, respectively. This study also points out that the values decrease in the single-family houses than in apartments which may have been caused by building characteristics (e.g., less exposed facades, sharing internal walls in case of apartments) but also by the occupant′s behavior related to the selection of the heating set-point (e.g., the elderly living mainly in apartments prefer slightly higher temperatures). Such conclusions can be deductive for our study. Because it is not possible to say whether higher or lower values of temperature were achieved in renovated or non-renovated houses.

In our study the mean relative humidity ranges from 36.0% to 64.0% in single family houses and from 34.1% to 68.0% in apartments. Standard deviation ranges from 0.3 to 1.4. According to study [18] the mean relative humidity was higher in the single-family houses than in the apartments (34% vs. 31%). These values correspond with the required range of 30%–70%.

### 3.2. Total Volatile Organic Compounds

In Table 3, there are TVOC concentrations measured in the selected houses. As can be seen, the mean values of TVOC concentrations ranged from 50 μg/m^3^ to 2610 μg/m^3^. The lowest mean level (50 μg/m^3^) was measured in house (House 6) with a maximum mean relative humidity. The recommended value (200 μg/m^3^) [36] for human exposure to TVOC was exceeded in 32% of all houses. However in 73% of houses with allowed smoking were observed with very high concentrations ranged from 206 to 2610 μg/m^3^. Study [37] ascertains that in the pre-occupancy stage, the median TVOC levels were low in two houses (less than 150 μg/m^3^) and high in the other houses (between 500 and more that 3000 μg/m^3^). In study [38] the indoor total VOC levels were fairly low (1283 μg/m^3^) compared to other studies (210–6000 μg/m^3^) [39]. As noted above the 157 single-family houses and 148 apartments were monitored from the occurrence of TVOC concentrations too [18]. The mean TVOC concentration in single family houses was higher (306 μg/m^3^) than in apartments (174 μg/m^3^). This study takes a note that a previous study [40] conducted more than ten years ago found higher concentrations of 388 μg/m^3^ in single-family houses and 317 μg/m^3^ in apartments. Very high levels of TVOC concentrations in family houses were recorded in our study.

### 3.3. Particulate Matters

Box plot of PM_2.5_ concentrations in selected houses is shown in Figure 1. Mean concentrations of PM_2.5_ ranged from 16.80 µg/m^3^ to 30.70 µg/m^3^. Similar results to our observation were ascertained in study [37], in which the mass concentrations of PM_2.5_ were always below 30 μg/m^3^ and ranged from 6 to 28 μg/m^3^. Escobedo et al (2014) [41] found that the houses using natural gas for cooking had average 24 h indoor concentration of 8.0 μg/m^3^ for PM_2.5_. Households using electricity had corresponding value of 4.7 μg/m^3^. This study confirms that only the homes with an indoor cigarette-smoking event during the two weeks prior to the survey had the highest concentration of 28 μg/m^3^. The average indoor PM_2.5_ concentration was determined to be 7.0 μg/m^3^ [41]. A comprehensive study [42] confirms that indoor pollutants such as cigarette smoking and cooking are a major source of indoor PM_2.5_ concentration and their impact is much greater than that infiltrated from outside. The following results are gained: (a) smoking led to an increase in the level of indoor concentration to as much as 1280 μg/m^3^, which took several hours to settle down; (b) indoor concentration in a room subjected to smoking was about 0.6 times higher than that of a control room; (c) cooking activities contributed to the PM_2.5_ concentration in the kitchen, to a level of 3000 μg/m^3^ within a short period of time; and (d) human activities such as walking, dressing and sweeping contributed to an increase of indoor concentration by about 33%.

Figure 2 is a box plot showing the levels of PM_10_ concentrations. Mean concentrations of PM_10_ ranged from 38.30 µg/m^3^ to 74.60 µg/m^3^. Huang et al. (2016) [43] conducted a case-control study with home inspection among 454 children in Shanghai, China. More than 70% of the child′s bedrooms had ≤75 μg/m^3^ PM_2.5_ and ≤150 μg/m^3^ PM_10_ [43]. The results of indoor air quality research in typical temperate zone in Australian homes are introduced in study of Molloy et al. (2012) [17]. This study points out that Summer/Autumn indoor PM_10_ (22.6 ± 9.0 μg/m^3^) was significantly higher than Winter/Spring indoor PM_10_ (18.3 ± 6.6 μg/m^3^). This study also suggests that heating was significantly correlated with PM_10_ which may be due to the use of wood heaters in some homes during the Winter/Spring period.

### 3.4. Noise Level

Figure 3 is a box plot showing the values of sound pressure level. The mean values of sound pressure level ranged from 29.8 dB(A) to 50.6 dB(A). In Macedonia, there are Regulations about the values of noise in working and auxiliary rooms, in that Regulations value is 50 dB(A) [44]. But living rooms noise should be limit value of 40 dB(A) was exceeded.

Study [37] shows that monitored bedrooms were generally the quietest (less than 30–33 dB(A)), except in two houses (up to 48 and 36 dB(A), respectively). Mean noise level in kitchens and drawing rooms was 53.58 and 55.67 dB(A)respectively in rural and urban houses in India [45]. Study of Ryu and Jeon [46] showed that noise sensitivity influenced the annoyance level caused by both indoor and outdoor noise. Bivariate analysis in study of Hammersen et al. (2016) revealed associations between high levels of noise annoyance and impaired mental health for all noise sources except air traffic [47].

### 3.5. Statistical Analysis

Further advantage of Permutational multivariate analysis of variance is that factor variables can be continuous but categorical as well. It reveals that we cannot conclude factors: Temperature, Relative humidity, Heating system and Year of construction. They have any effect on dependent variable TVOC, PM_2.5_, PM_10_. Table 4 shows the opposite case. Evidently the factor Smoke influences TVOC, PM_2.5_, and PM_10_ in the case of multivariate dependence. Nevertheless, Van der Waerden test reveals that we cannot conclude statistical dependence of Smoke on PM_10_ (similar situation is in other cases), only on TVOC and PM_2.5_ (Table 5).

Significance levels α are indicated with stars (asterisks). If the *p*-values are less than or equal to the significance level, then the outcome is said to be statistically significant (* significant at *p* < 0.05 = α).

**Figure 4:**
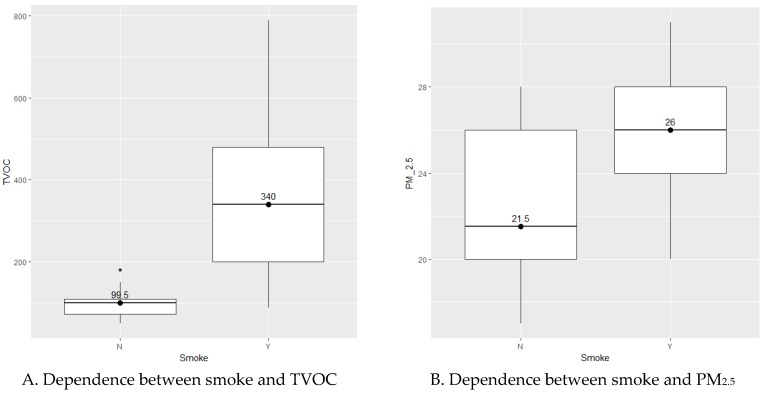
Box plots of significant factor smoke with medians. N, no; Y, yes.

**Figure 5:**
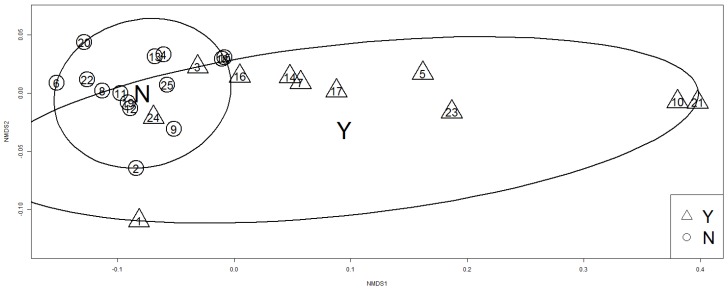
Non-metric multidimensional scaling visualizing the level of similarity for factor smoke. N, no; Y, yes.

For differences of TVOC and PM_2.5_ we can see it on box plots (Figure 4). In the case of Renovation factor the situation is more complicated. There is no evidence of influence concerning only itself. But PERMANOVA shows us the effect of interaction of Renovation and Smoke. This means hidden dependence. In the Figure 5 of NMDS are obviously two particular groups of houses differentiated by the factor Smoke. Evidently there is an intersection, which confirms previous ideas (interaction). Confidence areas (ellipses) show that we cannot include specific house in exactly one of the groups for 100%, but there is quite high probability to be in the group Smoke/Y.

## 4. Conclusions

This study has discussed the indoor air quality in 25 homes in Prilep, Republic of Macedonia during the winter period. Results were compared with other surveys performed over the world. Because of the limited set of houses, the findings cannot be generalized. Our results indicated that indoor relative humidity in homes meets the requirement of 30%–70%. The mean values of sound pressure level were high in most of the houses. The recommended value for TVOC (200 μg/m^3^) was exceeded in 32% of houses. Concentrations of PM_10_ were high in 64% of the investigated houses. This study shows that family house users are highly exposed to excessive noise, high concentrations of total volatile organic compounds as well excessive occurrence of particulate matters in the indoor air. The results of the present study are comparable to the results of other studies mentioned above. (Section 3). Some differences are related to different boundary conditions and the chosen methodology for measuring the environmental parameters.

By statistical analysis correlations between smoke and TVOC; smoke and PM_2.5_ as well as hidden dependence between renovation and smoke were found.

The study confirmed the high concentrations of the environmental parameters. General knowledge of indoor air quality in family houses is often very low and the occupants do not know that exposure to these pollutants has an impact on their health and comfort. Therefore, the indoor air quality needs to be investigated and people need to be informed of the possible health consequences. Long-term measurements of indoor environmental parameters need to be performed in order for these parameters to be generalized. This will be the objective of our future research work.

## Figures and Tables

**Table 1:** Basic information about investigated houses.

House	Year of Construction	Year of Renovation/ Heating System Changing	Living Room Area (m^2^)	Number of Inhabitants	Smoke	Heating System	Floor
1	1990	2007/2015	56	4	Y	CS—pellets	First
2	1990	2007/2015	33	2	N	CS—pellets	Ground
3	1980	-	30	4	Y	CS—wood	First
4	1980	-	18	2	N	CS—wood	Ground
5	1963	-	22	2	Y	WS	Ground
6	1965	-	18	4	N	EE	Fourth
7	1960	-	28	1	Y	EE	First
8	2005	-	45	4	N	HP	First
9	2005	-	40	4	N	HP	First
10	1970	-	27	4	Y	EE	Third
11	1967	-	34	4	N	EE and WS	First
12	1978	2013/-	27	4	N	CS—wood	First
13	1985	2003/-	24	2	N	EE	First
14	1985	2003/-	38	4	Y	EE and WS	Ground
15	1964	-	17	5	N	EE	Fourth
16	1975	-	35	2	Y	EE	First
17	1975	-	28	4	Y	CS—wood	First
18	1978	-	34	3	N	CS—pellets	First
19	1978	-	18	2	N	CS—pellets	Ground
20	1978	-	34	4	N	CS—wood	First
21	1978	-	18	2	Y	CS—wood	Ground
22	1982	-	26	4	N	CS—wood	First
23	1968	-	19	3	Y	EE	First
24	1972	2000/-	28	4	Y	CS—pellets	Second
25	1972	2000/-	20	1	N	CS—pellets	First

CS, Central System; Y, yes; N, no; EE, Electric Energy; WS, Wood Stove; HP, Heat Pump.

**Table 2:** Data about instruments used during IAQ monitoring.

No.	Measuring Instrument	Model	Technical Data
1	Temperature meter	AR847	Temperature: −10–+50 °C, accuracy: ±1 °C
2	Humidity meter	AR847	Humidity: 5% RH–98% RH, accuracy: ±3.5% RH
3	TVOC gas detector	KT603	detection range: 0–2000 ppm, resolution: 1000 ppb accuracy: ≤±3%
4	Nephelometer	TURNKEY DustMate kit	PM_1_; PM_2.5_; PM_10_; TSP measuring range: 0–6000 mg/m^3^ accuracy: ± 0.01 mg/m^3^
5	Sound level meter	SL-5868P	noise: 30–130 dB frequency from 20 to 12,500 Hz accuracy: ±1 dB

**Table 3:** Total volatile organic compounds.

House	Mean (μg/m^3^)	Standard Deviation	Min (μg/m^3^)	Max (μg/m^3^)	Median (μg/m^3^)	25.Percentil (μg/m^3^)	75.Percentil (μg/m^3^)
1	88	0.03	85	94	86	85	91
2	70	0.01	69	72	71	69	72
3	206	0.05	200	210	210	200	210
4	109	0.03	100	110	110	110	110
5	789	0.06	780	800	790	785	790
6	50	0.00	49	50	50	50	50
7	360	0.00	360	360	360	360	360
8	80	0.00	79	80	80	79	80
9	110	0.00	110	111	110	110	111
10	2610	0.00	2610	2610	2610	2610	2610
11	90	0.00	90	91	90	90	91
12	100	0.00	100	101	100	100	101
13	100	0.00	100	101	100	100	101
14	340	0.00	340	340	340	340	340
15	180	0.00	180	181	180	180	181
16	200	0.00	199	200	200	200	200
17	480	0.00	480	481	480	480	481
18	150	0.00	149	150	150	150	150
19	99	0.01	98	100	99	99	100
20	59	0.01	58	60	59	59	60
21	2580	0.00	2580	2581	2580	2580	2580
22	69	0.01	68	70	70	69	70
23	650	0.00	650	650	650	650	650
24	120	0.00	119	120	120	119	120
25	109	0.00	109	110	109	109	110

**Table 4:** Results of permutational multivariate analysis of variance.

Data	R^2^	Res. R^2^	Pseudo F	*p*-Value
Smoke and Renovation	0.36528	0.63472	4.0285	0.03398 *
Smoke	0.24212	0.75788	8.0108	0.00050 *
Renovation	0.05880	0.94117	0.1594	0.15940

* significant at *p* < 0.05 = α.

**Table 5:** Results of the Van der Waerden test for univariate cases.

Data	Van der Waerden Chi-Squared	df	*p*-Value
TVOC and Smoke	12.31	1	0.00045 *
PM_2.5_ and Smoke	4.17	1	0.04109 *
PM_2.5_ and Renovation	0.10	1	0.75350
PM_10_ and Smoke	2.74	1	0.09785
TVOC and Renovation	0.81	1	0.36840
PM_10_ and Renovation	3.59	1	0.05832

* significant at *p* < 0.05 = α.

## Abbreviations

The following abbreviations are used in this manuscript:

IEQ	Indoor environmental quality
OEQ	Overall Environmental Satisfaction
IAQ	Indoor Air Quality
PM	Particulate Matter
TSP	Total Suspended Particulate Matter
TVOC	Total Volatile Organic Compound
WHO	World Health Organization
RH	Relative Humidity
ppb	Parts Per Billion
R	Permutation of Residuals
F	F-test
P	Significance level
NMDS	Non-metric multidimensional scaling
